# Supplementary material for: RadA, a Key Gene of the Circadian Rhythm of Escherichia coli
Source: Int J Mol Sci. 2022 May 30;23(11):6136. doi: 10.3390/ijms23116136 (PMC9181324; doi:10.3390/ijms23116136)
Supplement: Supplementary file 1 [file ijms-23-06136-s001.zip › ijms-1735366-supplementary.pdf]

Supplementary Materials

A.

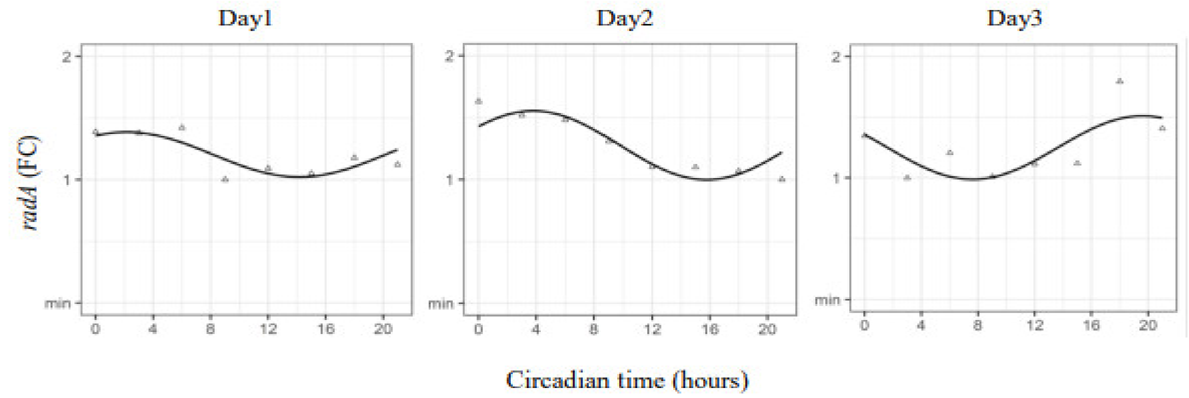

B.

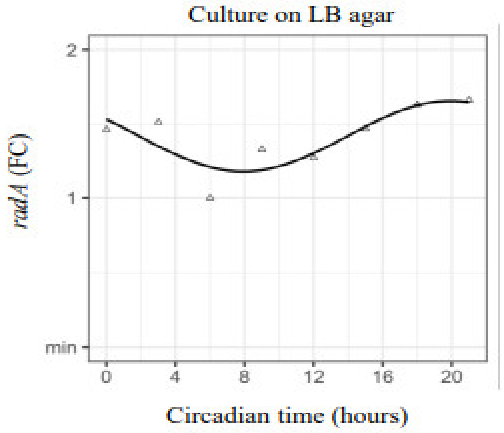

Figure S1. Circadian expression of *radA* in *E. coli* cultured on LB agar and persistence overtime. (**A**) Bacteria were spotted on blood agar with 7. 10^5 bacteria per point and incubated at 37°C for 24 hours. Colonies were then collected every 3 hours during 3 consecutive days. The expression of the *radA* gene was evaluated by q-RTPCR and expressed as fold change (FC) using the $2^{-\Delta\Delta Ct}$ formula. The triangles correspond to the raw data without Cosinor transformation; the sinusoids represent the expression of the genes over time after adjustment of the values by the Cosinor model. Statistical analysis was performed using the cosine function in R studio (the results are representatives of three experiments). (**B**) Bacteria were spotted on LB agar with 7. 10^5 bacteria per point and incubated at 37°C for 24 hours. Colonies were then collected every 3 hours for 24 hours. The expression of *radA* was evaluated by q-RTPCR and expressed as fold change (FC) using the $2^{-\Delta\Delta Ct}$ formula. The triangles correspond to the raw data without Cosinor transformation; the sinusoids represent the expression of the genes over time after adjustment of the values by the Cosinor model. Statistical analysis was performed using the cosine function in R studio (the results are representatives of three experiments).

A.

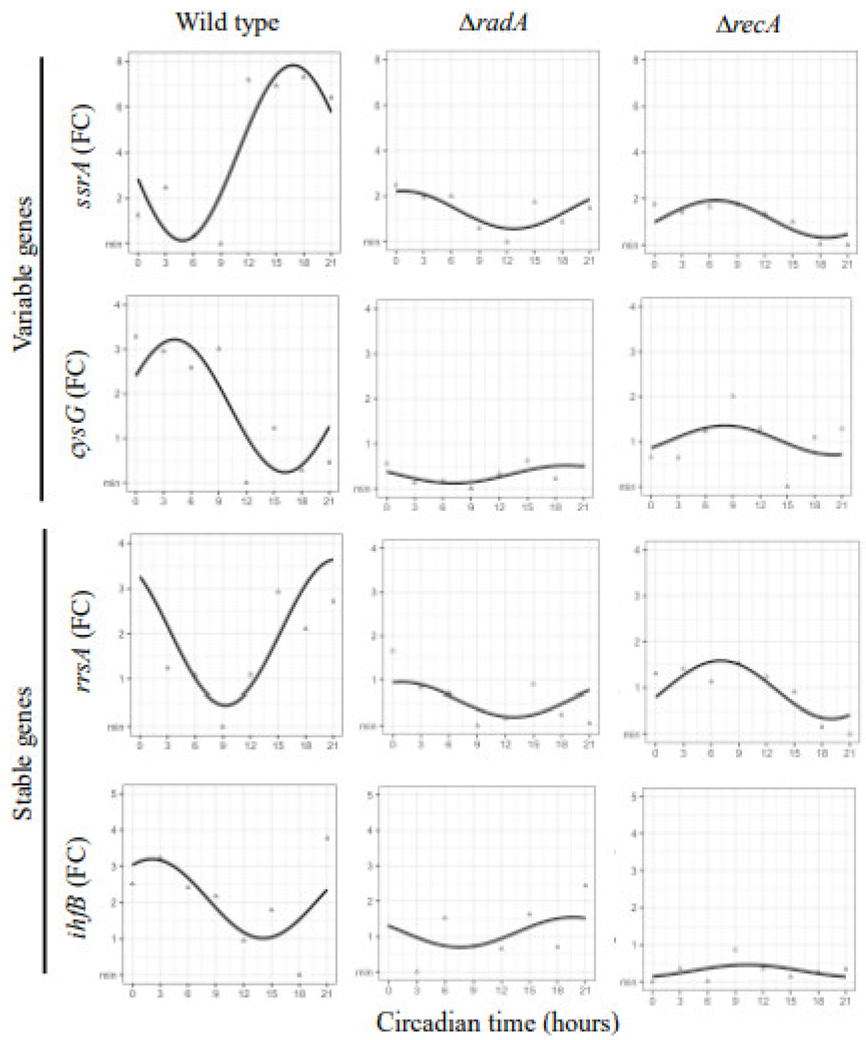

B.

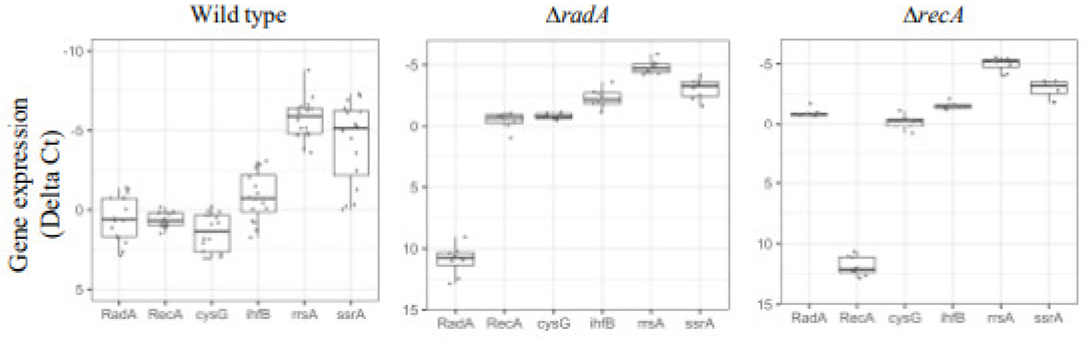

Figure S2. Circadian expression of variable and stable genes in *E. coli*. Bacteria were spotted on blood agar with 7. 10^5 bacteria per point and incubated at 37°C for 24 hours. Colonies were then collected every 3 hours for 24 hours. (**A**) The expression of the variable genes (*ssrA* and *cysG*) and the stable genes (*rrsA* and *ihfB*) was evaluated by q-RTPCR and expressed as fold change (FC) using the $2^{-\Delta\Delta Ct}$ formula. The triangles correspond to the raw data without Cosinor transformation; the sinusoids represent the expression of the genes over time after adjustment of the values by the Cosinor model. Statistical analysis was performed using the cosine function in R studio (the results are

representatives of three experiments). (**B**) The expression of the genes (*radA*, *recA*, *ssrA*, *cysG*, *rrsA* and *ihfB*) was evaluated by q-RTPCR and expressed in ΔCt. The results are represented as boxplot (median of the expression over 24h ± interquartile).

Table S1. Rhythmic parameters (MESOR, amplitude and acrophase) of *radA* during 3 days culture.

Gene	Days	MESOR	CI 95%	*p* Value	Amplitude	CI 95%	*p* Value	Acrophase	CI 95%	*p* value	Signifiant
radA	1	1.20	(1.12 ; 1.28)	**<0.001**	0.18	(0.07 ; 2.20)	**0.001**	2.16	(0.00; 4.48)	**0.006**	**Yes**
	2	1.27	(1.17 ; 1.37)	**<0.001**	0.27	(0.13 ; 0.41)	**<0.001**	3.78	(1.86 ; 5.70)	**<0.001**	**Yes**
	3	1.24	(1.10 ; 1.39)	**<0.001**	0.26	(0.05 ; 0.46)	**0.01**	19.61	(16.65 ; 22.58)	**0.003**	**Yes**

Table S2. Rhythmic parameters (MESOR, amplitude and acrophase) of *radA* in bacteria cultivated in LB agar.

Gene	MESOR	CI 95%	*p* Value	Amplitude	CI 95%	*p* value	Acrophase	CI 95%	*p* value	Signifiant
radA	1.41	(1.32 ; 1.51)	**<0.001**	0.23	(0.10 ; 0.37)	**<0.001**	19 .92	(17.75 ; 22.09)	**<0.001**	**Yes**
